# Endothelial Membrane Remodeling Is Obligate for Anti-Angiogenic Radiosensitization during Tumor Radiosurgery

**DOI:** 10.1371/journal.pone.0012310

**Published:** 2010-08-19

**Authors:** Jean-Philip Truman, Mónica García-Barros, Matthew Kaag, Dolores Hambardzumyan, Branka Stancevic, Michael Chan, Zvi Fuks, Richard Kolesnick, Adriana Haimovitz-Friedman

**Affiliations:** 1 Department of Radiation Oncology, Memorial Sloan-Kettering Cancer Center, New York, New York, United States of America; 2 Laboratory of Signal Transduction, Memorial Sloan-Kettering Cancer Center, New York, New York, United States of America; 3 Department of Surgery, Memorial Sloan-Kettering Cancer Center, New York, New York, United States of America; 4 Department of Cancer Biology and Genetics, Memorial Sloan-Kettering Cancer Center, New York, New York, United States of America; 5 Wake Forest University School of Medicine, Winston-Salem, North Carolina, United States of America; Wayne State University School of Medicine, United States of America

## Abstract

**Background:**

While there is significant interest in combining anti-angiogenesis therapy with conventional anti-cancer treatment, clinical trials have as of yet yielded limited therapeutic gain, mainly because mechanisms of anti-angiogenic therapy remain to a large extent unknown. Currently, anti-angiogenic tumor therapy is conceptualized to either “normalize” dysfunctional tumor vasculature, or to prevent recruitment of circulating endothelial precursors into the tumor. An alternative biology, restricted to delivery of anti-angiogenics immediately prior to single dose radiotherapy (radiosurgery), is provided in the present study.

**Methodology/Principal Findings:**

Genetic data indicate an acute wave of ceramide-mediated endothelial apoptosis, initiated by acid sphingomyelinase (ASMase), regulates tumor stem cell response to single dose radiotherapy, obligatory for tumor cure. Here we show VEGF prevented radiation-induced ASMase activation in cultured endothelium, occurring within minutes after radiation exposure, consequently repressing apoptosis, an event reversible with exogenous C_16_-ceramide. Anti-VEGFR2 acts conversely, enhancing ceramide generation and apoptosis. *In vivo*, MCA/129 fibrosarcoma tumors were implanted in *asmase^+/+^* mice or *asmase^−/−^* littermates and irradiated in the presence or absence of anti-VEGFR2 DC101 or anti-VEGF G6-31 antibodies. These anti-angiogenic agents, only if delivered immediately prior to single dose radiotherapy, de-repressed radiation-induced ASMase activation, synergistically increasing the endothelial apoptotic component of tumor response and tumor cure. Anti-angiogenic radiosensitization was abrogated in tumors implanted in *asmase^−/−^* mice that provide apoptosis-resistant vasculature, or in wild-type littermates pre-treated with anti-ceramide antibody, indicating that ceramide is necessary for this effect.

**Conclusions/Significance:**

These studies show that angiogenic factors fail to suppress apoptosis if ceramide remains elevated while anti-angiogenic therapies fail without ceramide elevation, defining a ceramide rheostat that determines outcome of single dose radiotherapy. Understanding the temporal sequencing of anti-angiogenic drugs and radiation enables optimized radiosensitization and design of innovative radiosurgery clinical trials.

## Introduction

There is general interest in combining anti-angiogenesis therapy with conventional anti-cancer treatment, and clinical trials are underway testing a variety of agents[1]. The original concept was that anti-angiogenic treatment would act to “choke off” a growing tumor that has a burgeoning need for blood vessels to provide oxygen and nutrients[2]. Recent adaptations of this concept conceive anti-angiogenic therapy to act by two differing, though non-mutually exclusive, mechanisms. One postulates anti-angiogenesis prevents VEGF-dependent recruitment of endothelial precursors into nascent or damaged tumor vasculature[3], while the other proposes anti-angiogenic therapies “normalize” dysfunctional tumor vasculature thereby improving perfusion and drug delivery[3], [4]. Although the outcome of some clinical studies are consistent with either of these hypotheses[3], [4], to date anti-angiogenesis therapy has yielded only modest gains. It thus appears that while anti-angiogenesis may have potential impact in anti-cancer therapy, its mode of application has so far not been optimized, limiting its utility.

We recently reported that single dose radiotherapy induces a rapid wave of endothelial cell apoptosis in radioresponsive tissues, such as the gastrointestinal tract and tumors, that acts in concert with direct damage to tissue-specific stem cells to determine organ fate[5], [6], [7], [8]. We termed this event the vascular component of the tumor (or tissue) response, and showed that its abrogation in acid sphingomyelinase (ASMase)-, Bak-, or Bax-deficient mice resulted in resistance to single dose radiotherapy. We also showed [8] that Bax and Bak have non-redundant functional roles in the apoptotic response of the irradiated intestinal endothelium. Pre-treatment with angiogenic basic fibroblast growth factor (bFGF) mimicked the *asmase^−/−^* phenotype[5]. Endothelial cells preferentially manifest 20-fold enrichment of a non-lysosomal secretory form of ASMase[9] that renders them particularly vulnerable to radiation-induced ASMase-mediated generation of the pro-apoptotic second messenger ceramide[10], and evidence indicates ceramide-mediated apoptosis is causative of the vascular component of tumor response to single dose radiotherapy[10]. While these studies employed genetic inactivation of endothelial apoptosis to argue that tissue damage occurred by a combined effect of direct damage to stem cells coupled to vascular dysfunction, here we test the hypothesis that anti-angiogenesis therapy might act in converse, targeting the vascular component, de-repressing this system in order to radiosensitize. The present studies show that the ceramide level serves as a radiation rheostat that regulates the balance between endothelial cell survival and death, and ultimately tumor response. Utilization of anti-angiogenic drugs based on the principle of enhancing ceramide signaling resulted in conversion of tumor growth delay to tumor cure after single dose radiotherapy.

## Results

### VEGF and bFGF inhibit radiation-induced apoptosis via repression of ASMase activation

**Figure 1:**
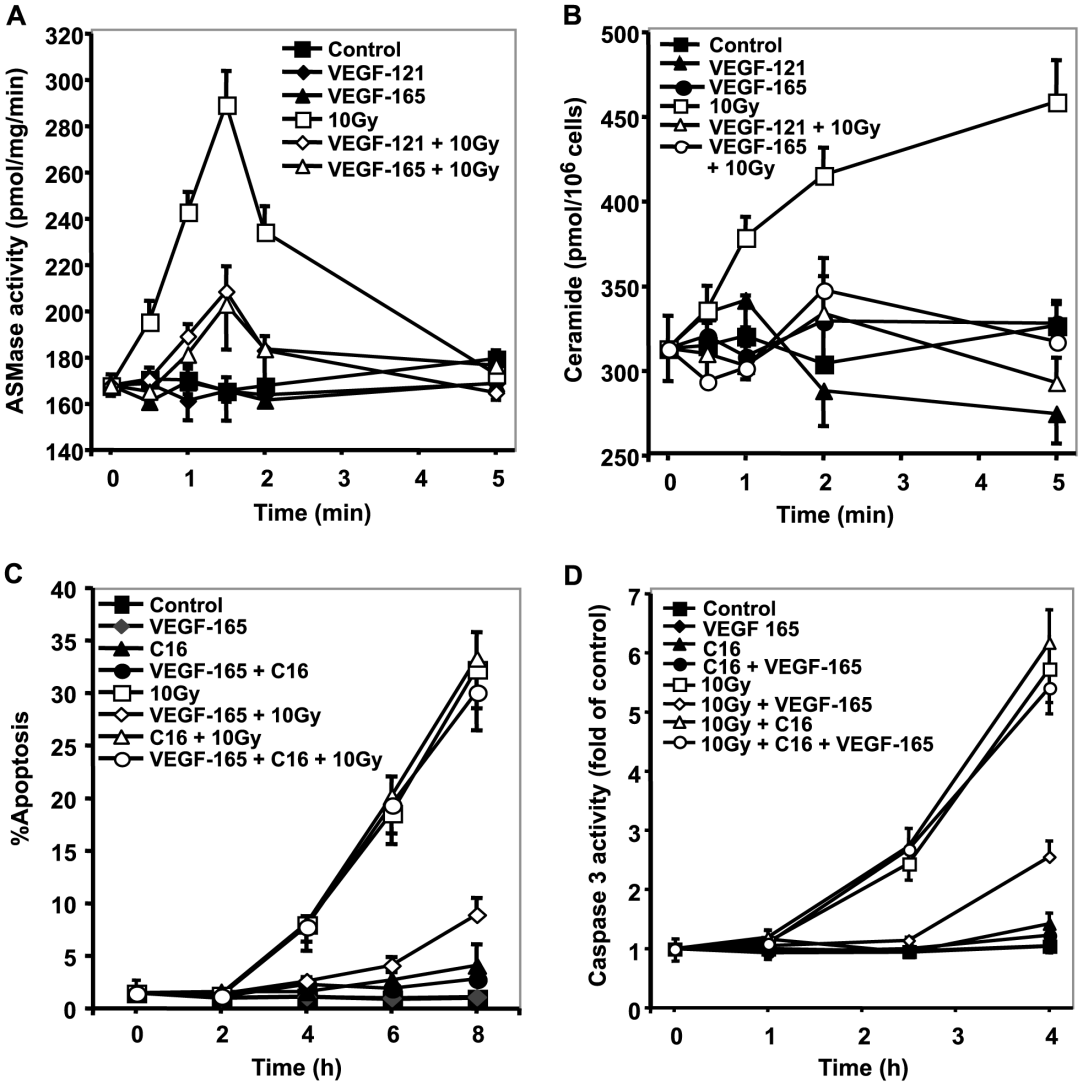
VEGF inhibits radiation-induced apoptosis via repression of ASMase activation. (**A**) ASMase activity following 10 Gy is inhibited by VEGF pre-treatment. Irradiated BAEC samples were collected at the indicated times and ASMase activity measured by quantifying conversion of [^14^C]sphingomyelin to the product [^14^C]phosphocholine. Data (mean±s.d.) represent duplicate determinations from 2 experiments. (**B**) Radiation-induced ceramide generation is inhibited by VEGF pre-incubation. VEGF (1 ng/ml) was added 15 min before irradiation. Ceramide was quantified at the indicated times by the diacylglycerol kinase assay. Data (mean±s.d.) represent triplicate determinations from 2 experiments. (**C**, **D**) VEGF pre-treatment inhibits radiation-induced apoptosis. C_16_-ceramide (C_16_, 1 µM) was added 30 min prior to irradiation. Samples were fixed in 10% paraformaldehyde prior to *bis*-benzimide staining. For apoptosis quantification, data (mean±s.d) represent duplicate determinations of at least 400 *bis*-benzimide stained nuclei counted from 2 experiments. Caspase 3 activity was measured by quantitation of the luminescence of cleaved DEVD-AMC substrate. Data (mean±s.d.) represent duplicate points from 3 experiments.

VEGF and bFGF inhibit radiation-induced endothelial apoptosis *in vitro* and *in vivo* [11], [12], although the mechanism remains only partially known. While bFGF was shown to inhibit endothelial apoptosis via repression of ASMase-mediated ceramide elevation[13], a similar effect of VEGF has not been reported. Here we show that the increase in ASMase activity in bovine aortic endothelial cells (BAEC), which occurs almost immediately after 10 Gy from a baseline of 168±6 nmol/mg/min to peak at 289±15 nmol/mg/min at 1.5 min (**Figure 1A**; p<0.005), was abrogated by pre-treatment with VEGF-165, VEGF-121 (**Figure 1A**; p<0.005 each vs. radiation alone) or bFGF (**Figure S1A**; p<0.005 vs. radiation alone). Concomitant radiation-induced increases of neutral sphingomyelinase (NSMase) or ceramide synthase activity were not detected (not shown). Whereas ASMase translocation to the external leaflet of the plasma membrane precedes ASMase-mediated sphingomyelin hydrolysis to ceramide in diverse stress systems[14], we show that in BAEC surface ASMase protein, detected by FACS (see Supplementary Methods S1), increased by 2 min post 10 Gy from a baseline in 10±1% to a maximum of 29±1% of the total cell population (**Figure S1B**; p<0.01 vs. non-irradiated control), and was almost completely inhibited by 10 min pre-treatment with 1 ng/ml bFGF (p<0.01 vs. radiation alone). Similarly, pre-treatment with 2 ng/ml VEGF-165 or VEGF-121 almost completely inhibited radiation-induced ASMase translocation (not shown), resulting in inhibition of radiation-induced ceramide elevation (**Figure 1B**; p<0.01 for each VEGF plus radiation vs. 10 Gy alone), thus mimicking bFGF (**Figure S1C**; p<0.01). Inhibition of radiation-induced ceramide elevation by VEGF and bFGF was also observed in human coronary aortic endothelial cells (HCAEC) (**Figure S2**).

As anticipated, based on prior studies[11], ceramide repression by VEGF and bFGF was followed by inhibition of radiation-induced endothelial apoptosis (assessed by *bis*-benzimide staining) in VEGF-165- (**Figure 1C**), VEGF-121- (**Figure S3A**) or bFGF- (**Figure S4**) pre-treated BAEC (p<0.01 each vs. 10 Gy alone at all times from 4–8 h) or HCAEC (**Figure S5**; p<0.01 each vs. 20 Gy alone at 9–12 h). Similar anti-apoptotic protection was demonstrated using the caspase 3 activity assay, which increased 5.7-fold of control by 4 h after 10 Gy, reduced to 2.7-fold by VEGF-165 (p<0.05, **Figure 1D**), 2.6-fold by VEGF-121 (**Figure S3B**; p<0.05), and nearly abrogated by bFGF (**Figure S6**, p<0.001 vs. 10 Gy).

To confirm the critical role of ceramide in signaling apoptosis in this cell system, a small amount of long-chain natural C_16_-ceramide (1 µM) was added 30 min prior to irradiation, with or without pre-treatment with VEGF or bFGF. This amount of C_16_-ceramide alone did not confer apoptosis for as long as 8 h, and did not significantly increase radiation-induced BAEC apoptosis (32% compared with 33% at 8 h). In contrast, this non-apoptotic dose of C_16_-ceramide bypassed the anti-apoptotic effect of VEGF-165 (**Figure 1C**), VEGF-121 (**Figure S3A**), and bFGF (**Figure S4**), restoring radiation-induced apoptosis to a level comparable to 10 Gy alone. C_16_-dihydroceramide (1 µM), which differs from ceramide by a double bond in the sphingoid base backbone, had no effect on VEGF and bFGF inhibition of radiation-induced BAEC apoptosis (data not shown). Increasing bFGF up to 8 ng/ml did not alter restitution of radiation-induced apoptosis by C_16_-ceramide (**Figure S7**). Similarly, C_16_-ceramide bypassed VEGF and bFGF inhibition of radiation-induced apoptosis in HCAEC (**Figure S5**). Taken together, these studies indicate inhibition of ASMase-mediated ceramide elevation as the mechanism of the anti-apoptotic effect of VEGF and bFGF in endothelial cells.

### VEGF regulates a pro-apoptotic ceramide rheostat *via* ASMase repression

**Figure 2:**
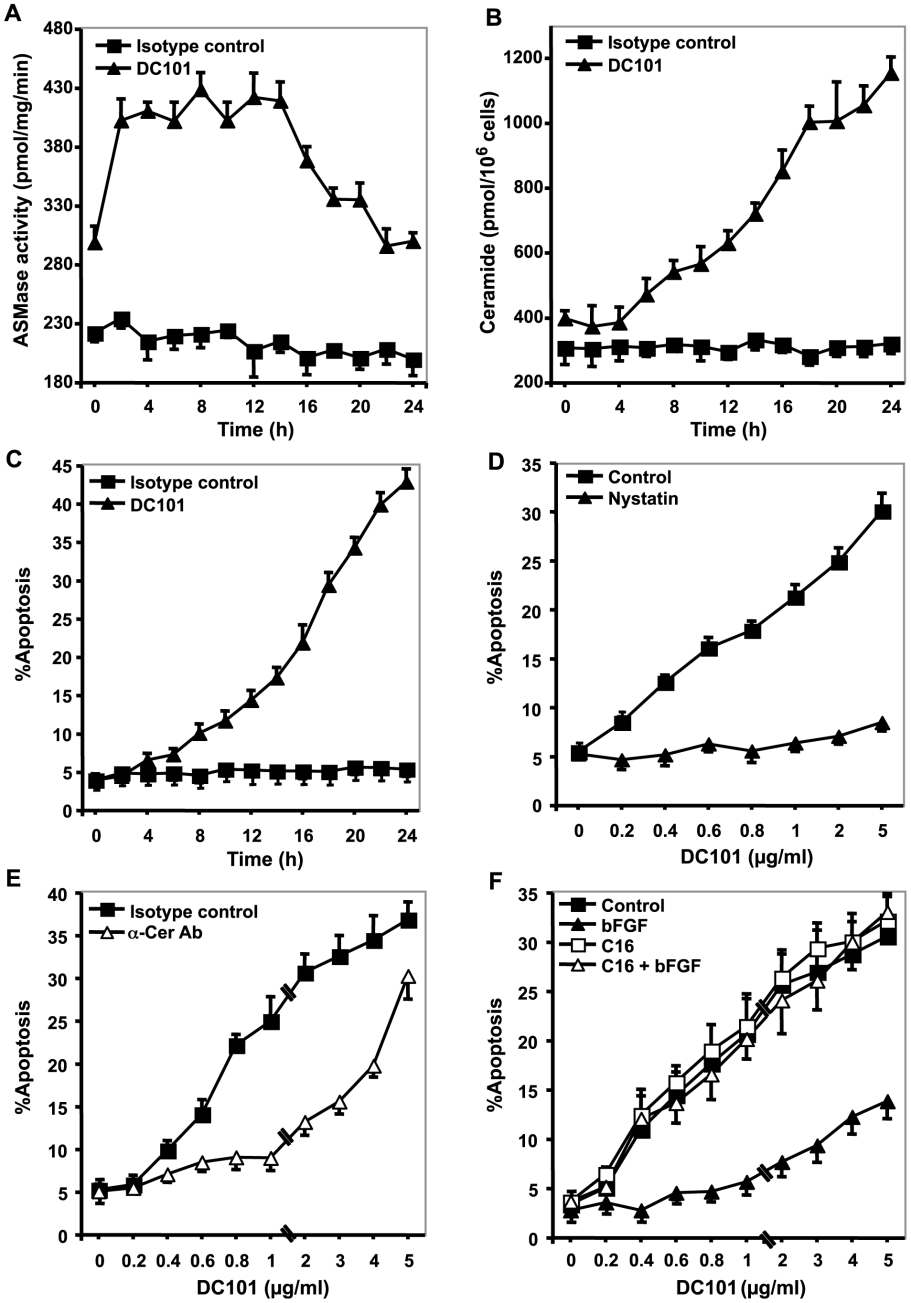
VEGF regulates a pro-apoptotic ceramide rheostat *via* ASMase repression. (**A**) ASMase activation in response to DC101. A19 BAEC were incubated with 5 µg/ml DC101 and ASMase activity was measured every 2 h for 24 h by quantifying conversion of [^14^C]sphingomyelin to the product [^14^C]phosphocholine as in **Fig. 1A**. Data (mean±s.d.) represent triplicate determinations from 2 experiments. (**B**) DC101 (5 µg/ml) induces ceramide elevation. Ceramide was quantified by the diacylglycerol kinase assay as in **Fig. 1B** at the times indicated. Data (mean±s.d.) represent triplicate determinations from 2 experiments. (**C**) DC101 induces apoptosis. DC101(5 µg/ml) was added and cells collected every 2 h, fixed and apoptosis quantified using *bis*-benzimide method as in **Fig. 1C**. (**D**) Disruption of GEMs inhibits DC101-induced apoptosis. Nystatin (30 µg/ml) was added 30 min prior to the addition of escalating doses of DC101 and apoptosis quantified after 24 h as in **Fig. 1C**. (**E**) Anti-ceramide antibody MAS0020 inhibits apoptosis induction by DC101. MAS0020 was added at 250 ng/ml 30 min prior to the addition of increasing concentrations of DC101. After 24 h, apoptosis was quantified as in **Fig. 1C**. (**F**) bFGF inhibits DC101-induced apoptosis by inhibition of ceramide generation. bFGF (1 ng/ml) and C_16_-ceramide (1 µM) were added as detailed in **Fig. 1C**, and apoptosis was quantified after 24 h.

To further explore the modulation of the pro-apoptotic function of ceramide by VEGF, we employed the commonly used VEGFR2 antagonistic monoclonal antibody DC101[15]. Initial studies showed that A19 BAEC used here, like other endothelial cells[16], [17], [18], synthesize and secrete VEGF into the culture medium, predominantly biologically-active VEGF-165, and small amounts of VEGF-121 (data not shown). A maximal dose of 5 µg/ml DC101 induced a rapid increase of ASMase activity in BAEC to 402±18 nmol/mg/min (**Figure 2A**; p<0.005 vs. control) and subsequently increased ceramide mass (p<0.001 vs. control at ≥4 h, **Figure 2B**), peaking at 1003±50 pmol/10^6^ cells at 18 h. Ceramide elevation was DC101 dose-dependent over the range of 0.4–1 µg/ml, inhibitable by bFGF (**Figure S8**) within the range of 1–8 ng/ml (**Figure S9**). DC101 (5 µg/ml) also induced apoptosis (**Figure 2C**), increasing steadily to a peak of 43±2% at 24 h (p<0.001 vs. control).

The pivotal role of ceramide in induction of endothelial apoptosis by DC101 was supported by disrupting pre-formed plasma membrane glycosphingolipid-enriched domains (GEMs), the putative site of ceramide generation after ASMase activation[19], [20]. Pre-treatment (30 min) with the cholesterol binding agent nystatin (30 µg/ml) abolished the apoptogenic effect of DC101 (**Figure 2D**) at all doses up to 5 µg/ml, significantly reducing apoptosis from 30±2% to 9±1% (p<0.005). Furthermore, the anti-ceramide antibody MAS0020, which binds ceramide on the cell surface[21], significantly inhibited DC101-induced apoptosis up to 1 µg/ml (**Figure 2E**; p<0.01), exhibiting a dose modifying factor within the range of 4–5, a large effect. Furthermore, bFGF inhibition of the pro-apoptotic effect of DC101 was reversible by C_16_-ceramide (**Figure 2F**). Thus three independent approaches to inactivating ceramide, bFGF/VEGF inactivation of ASMase translocation, nystatin disruption of GEMs, and binding ceramide on the cell surface with an anti-ceramide antibody, all show ceramide signaling is critical to the pro-apoptogenic effect of DC101.

### Anti-angiogenic treatment (DC101) radiosensitizes via ASMase activation

**Figure 3:**
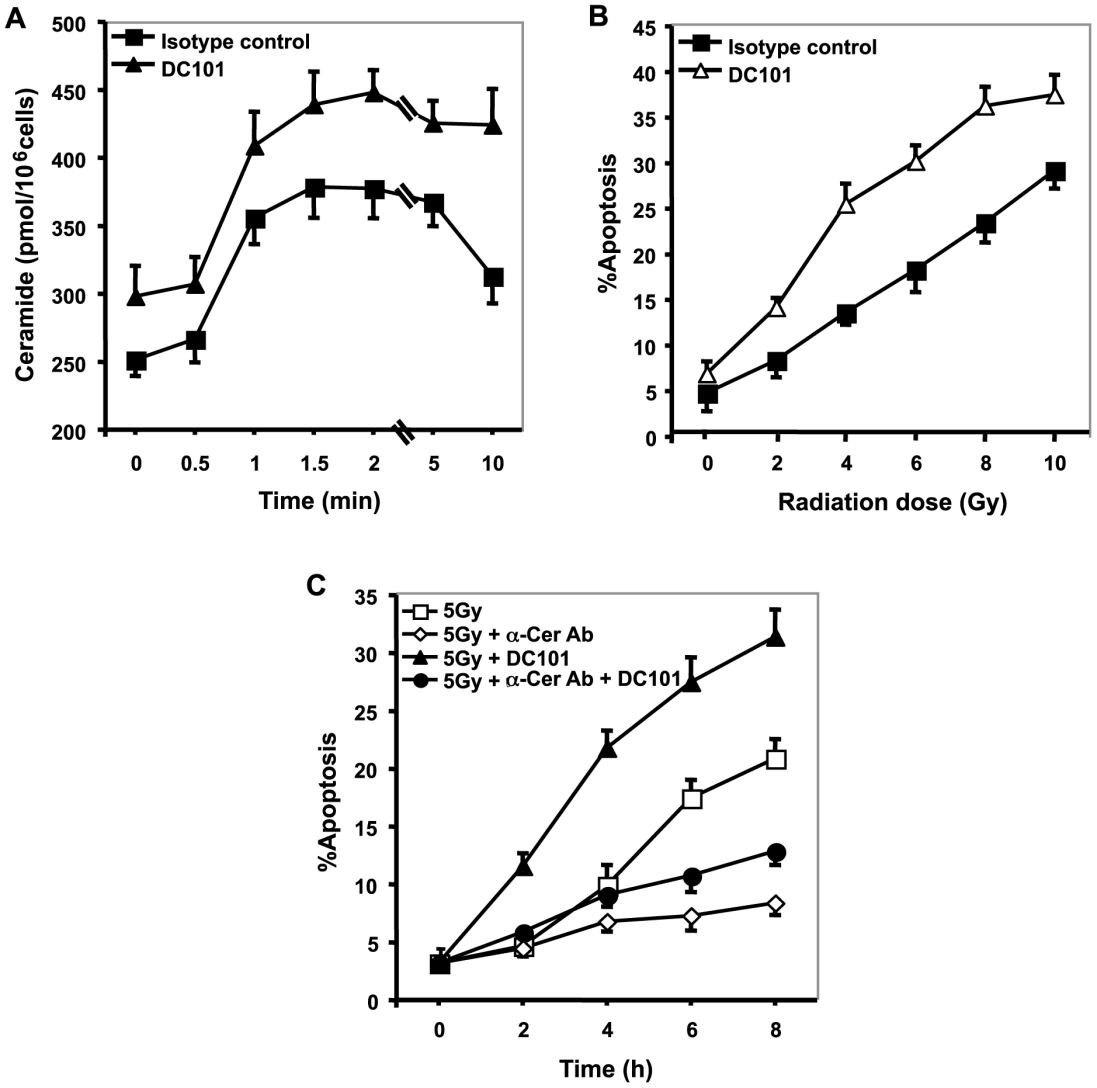
Anti-angiogenic treatment (DC101) radiosensitizes via ASMase activation. (**A**) DC101-pre-treatment increases radiation-induced ceramide elevation. Cells were pre-treated for 16 h with 0.2 µg/ml DC101, irradiated at 8 Gy, and ceramide was measured by diacylglycerol kinase assay as in **Fig. 1B**. Data (mean±s.d.) represent triplicate determinations from 2 experiments. (**B**) DC101 increases radiation-induced apoptosis. Cells were pre-incubated for 16 h with 0.2 µg/ml DC101. At 8 h post-irradiation, apoptosis was quantified using *bis*-benzimide staining. (**C**) Anti-ceramide antibody inhibits DC101-enhanced radiation-induced apoptosis. BAEC were treated with 350 ng/ml MAS0020 15 min prior to 0.2 µg/ml DC101, and after 16 h irradiated at 5 Gy. Apoptosis was quantified 8 h post-irradiation by *bis*-benzimide staining. Data in (**2C–F, 3A, and 3C**) represent means±s.d. of duplicate determinations from at least 400 scored nuclei from 2 separate experiments.

We also tested combining DC101 with radiation on ceramide elevation and apoptosis in BAEC. For these studies, DC101 was titrated to a dose of 0.2 µg/ml that yielded no significant apoptosis alone. When combined with radiation, 0.2 µg/ml DC101 significantly enhanced radiation-induced ceramide elevation (p<0.005 between 2–8 Gy), manifested as a higher peak and a slower decay in ceramide levels following irradiation (**Figure 3A**), and increased radiation-induced apoptosis at all radiation doses up to 10 Gy (**Figure 3B**), providing a dose-modifying factor of 2.0±0.3. This combined effect was attenuated by 350 ng/ml of the MAS0020 anti-ceramide antibody, which reduced the apoptosis induced by 5 Gy plus 0.2 µg/ml DC101 at 8 h from 32±2.3% to 13±1% (**Figure 3C**; p<0.001 vs. 5 Gy+DC101), indicating the centrality of ceramide in initiating radiation-induced apoptosis in BAEC. Similar responses were observed in HCAEC (**Figure S10**). The observation that VEGF does not inhibit apoptosis if ceramide elevation is not suppressed (as upon exogenous ceramide addition), nor do anti-angiogenics sensitize if ceramide function is abrogated (as by treatment with anti-ceramide antibody), implies a fundamental role for a ceramide rheostat in radiation-induced endothelial apoptosis.

### ASMase is required for radiosensitization by anti-VEGF effectors *in vivo*

**Figure 4:**
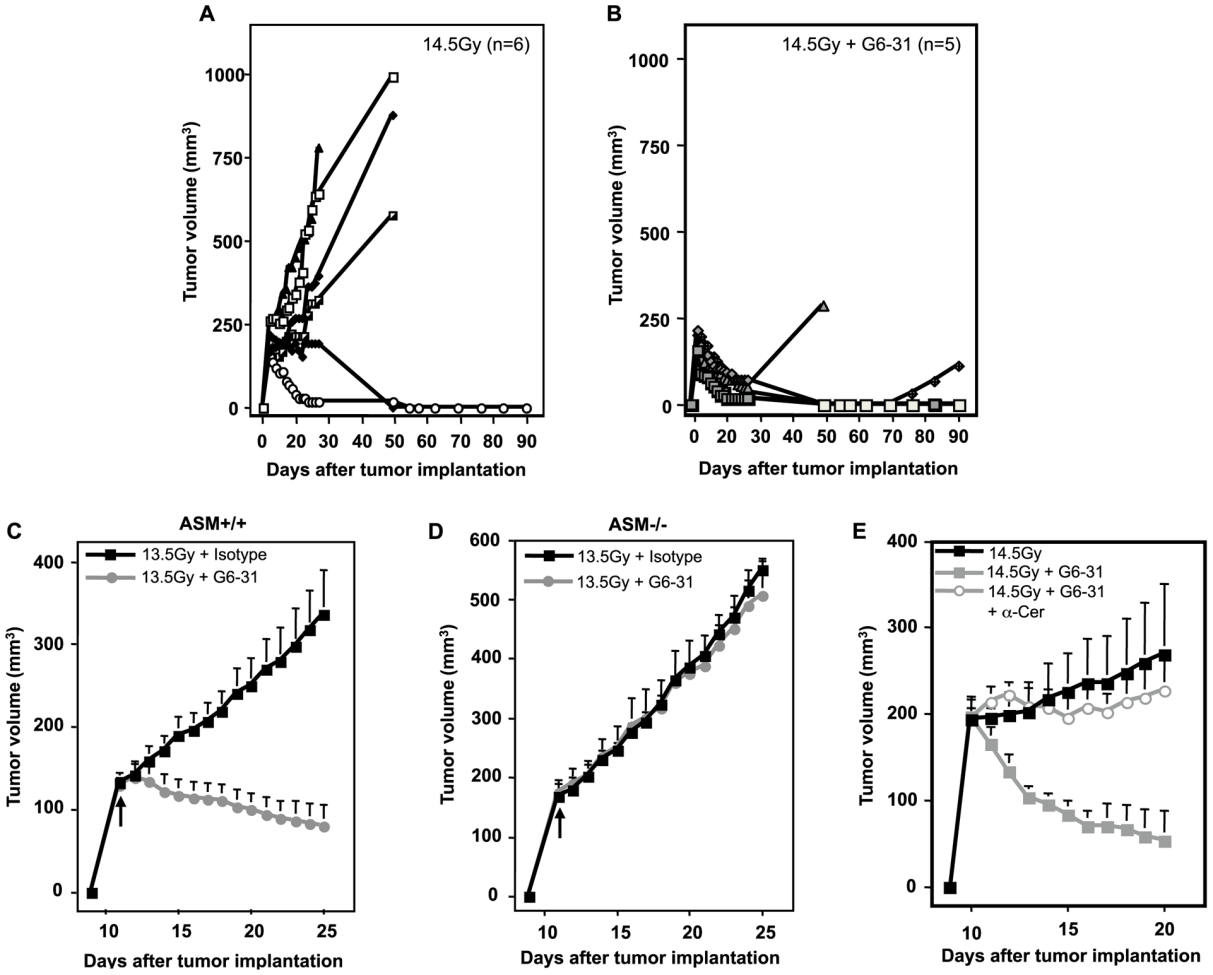
ASMase is required for anti-VEGF (G6–31) mAb radiosensitization *in vivo*. (**A** and **B**) Impact of 1 h pre-treatment with G6–31 (5 mg/kg) on tumor response to 14.5 Gy. N equals number of animals per group. (**C** and **D**) Impact of a 1 h pre-treatment with G6–31 (5 mg/kg) on tumor response to 13.5 Gy using tumor xenografts grown in *asmase^+/+^* (left panel) or *asmase^−/−^* littermates (right panel). No tumor growth delay was observed in tumors growing in ASMase deficient mice after 13.5 Gy pre-treated with G6–31 as compared to tumors growing in the *asmase^+/+^* littermates. Data (mean±s.e.m.) are collated from 5 animals per group. Arrows indicate the day of treatment. (**E**) Impact of anti-ceramide treatment on G6–31-enhanced radiation-response. G6–31 (5 mg/kg) was injected i.v. 1 h before 14.5 Gy irradiation. Anti-ceramide Ab MAS0020 (25 µg) was injected i.v. immediately before irradiation. Anti-ceramide treated mice displayed a significantly reduced tumor growth delay. Data (means ± s.e.m.) were collated from 4 mice per group.

As the cell culture studies indicated radiation and anti-angiogenic reagents act in concert to increase endothelial apoptosis via the ceramide rheostat, we tested whether anti-VEGF Ab G6-31 and anti-VEGF receptor Ab DC101 similarly amplify the vascular component of tumor response *in vivo*. We employed the mouse MCA/129 fibrosarcoma, previously shown by us to engage the vascular component in response to single dose radiotherapy, with 45–50% local tumor cures achieved with a single dose of 15 Gy[6]. In **Figure 4A** a single dose of 14.5 Gy resulted in 2 of 6 (33%) tumors showing an initial complete response and apparent local tumor cure at 90 days post radiotherapy, while the remaining exhibited tumor growth delay or no response. Pre-treatment with G6–31 (5 mg/kg)[22] delivered intravenously 1 h prior to radiation, significantly radiosensitized tumor response, with all tumors demonstrating an initial complete response, and with 3 of 5 (60%) maintaining local cure at 90 days (**Figure 4B**; p<0.001 vs. radiation alone). G6–31 alone did not affect unirradiated tumor growth (not shown). Combining G6–31 with a lower radiation dose of 13.5 Gy, which by itself conferred minimal tumor growth delay but no local cures (not shown), also resulted in synergistic enhancement of tumor response, yielding 2 of 5 (40%) cures at 90 days, and the rest exhibiting growth delay of 27 days (not shown and **Figure 4C**; p<0.001 compared to radiation alone). To put these data in perspective, in total we have evaluated 75 MCA/129 fibrosarcomas that received 13.5 Gy+isotype control antibody without detecting a single local cure. It should be noted that MCA/129 fibrosarcomas growing in *asmase^−/−^* littermates were completely resistant to the radiosensitizing effects of G6–31 (**Figure 4D**). Additionally, anti-ceramide Ab MAS0020, injected immediately before irradiation, abrogated G6–31 radiosensitization, phenocopying the ASMase knockout mice (**Figure 4E**). These observations confirm that anti-angiogenic tumor radiosensitization *in vivo* requires ASMase-mediated ceramide generation.

**Figure 5:**
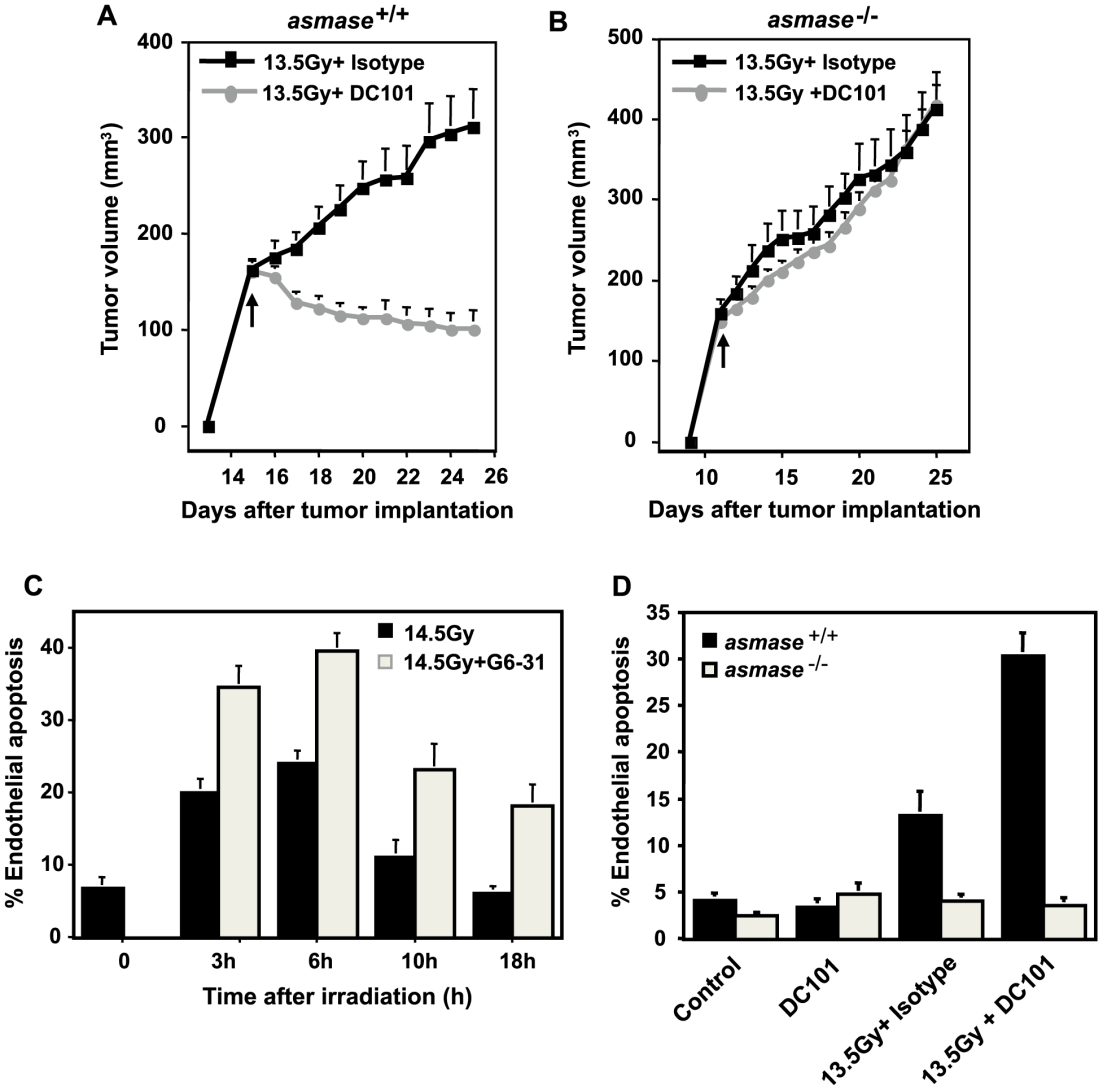
Role of ASMase in anti-angiogenic therapy. (**A**) Impact of a 1 h pre-treatment with DC101 (1600 µg/animal) on the tumor response to 13.5 Gy using MCA/129 fibrosarcomas grown in *asmase^+/+^* or (**B**) in *asmase^−/−^* littermates. Data (mean±s.e.m.) are collated from *asmase^+/+^* control mice (n = 7), DC101-treated *asmase^+/+^* mice (n = 9), and *asmase^−/−^* mice (n = 10 each). (**C**) Quantification of the G6–31 effect on radiation-induced endothelial cell apoptosis. Endothelial cells (mean±s.e.m.) from cross sections of 14.5 Gy-irradiated MCA/129 fibrosarcomas were stained with both an endothelial cell specific Ab (MECA-32) and TUNEL. Data was collated from 20 fields from 1 of 2 similar experiments employing 2 animals per group at the times shown. (**D**) Quantification of the effect of DC101 on 13.5 Gy radiation-induced endothelial cell apoptosis. Data (mean±s.e.m.) represent TUNEL-positive endothelial cells collated from 20 fields from 1 of 2 similar experiments employing 2 animals per group.

Similar radiosensitization was observed following pre-treatment with DC101. While 1600 mg DC101 per 25 g mouse alone did not affect unirradiated tumor growth (not shown), its intravenous delivery 1 h prior to 13.5 Gy significantly radiosensitized the tumor (**Figure 5A**), yielding 2/9 cures for at least 90 days and a mean of 18 days growth delay in the 7 remaining mice (not shown). As with G6–31, MCA/129 fibrosarcoma grown in *asmase^−/−^* littermates were completely resistant to 13.5 Gy±DC101 (**Figure 5B**), and anti-ceramide MAS0020 antibody injected immediately before irradiation abrogated the radiosensitizing effect of DC101 (not shown). Similarly, B16 melanoma, previously reported to engage a vascular component in response to single dose radiotherapy[6], is radiosensitized by DC101 (**Figure S11**), but when implanted in *bak^−/−^* mice, which provides radioresistant vasculature [8], similarly exhibited resistance to DC101-induced radiosensitization.

To confirm that anti-VEGF or anti-VEGFR2 increased radiation-induced endothelial cell apoptosis *in vivo*, MCA/129 tumors were excised at 4–6 h post-irradiation and co-stained with an antibody to the endothelial selective cell surface marker CD34 (blue in **Figure S12**) and TUNEL for apoptosis (brown), as described[6]. Neither G6–31 (not shown), nor DC101 (**Figure S12**) conferred tumor endothelial apoptosis when delivered alone. Pre-treatment with G6–31, increased endothelial apoptosis at 6 h post 14.5 Gy from 24±1.7% to 39.5±2.5% **(** **Figure 5C**; p<0.001), and DC101 enhanced apoptosis 3-fold at 6 h after 13.5 Gy (**Figure 5D**, p<0.001 vs. radiation alone). In contrast, neither radiation dose in the presence or absence of anti-angiogenic drugs had an impact on endothelial cell apoptosis in tumors grown in *asmase^−/−^* mice (data not shown). FACS analysis of single cell suspensions of disaggregated MCA/129 fibrosarcomas grown in *asmase^+/+^* and *asmase^−/−^* mice revealed similar numbers of CD31+/VEGFR2+ endothelial cells (**Figure S13**), and immunohistochemistry revealed that the vasculature of fibrosarcomas grown in *asmase^−/−^* mice displays a normal pattern of VEGFR2 expression, and normal VEGFR2 tyrosine phosphorylation upon intravenous VEGF injection (data not shown). Hence failure to respond to DC101 in an *asmase^−/−^* background cannot be attributed to VEGFR2 function deficiency. Taken together, these observations are consistent with the mechanism of tumor radiosensitization by anti-VEGF effectors acting *via* amplification of the vascular component, specifically antagonizing VEGF that is generically present in tumors, generated by and secreted from tumor cells[23].

### Timing of anti-angiogenic treatment is critical for radiosensitizing the vascular component

**Figure 6:**
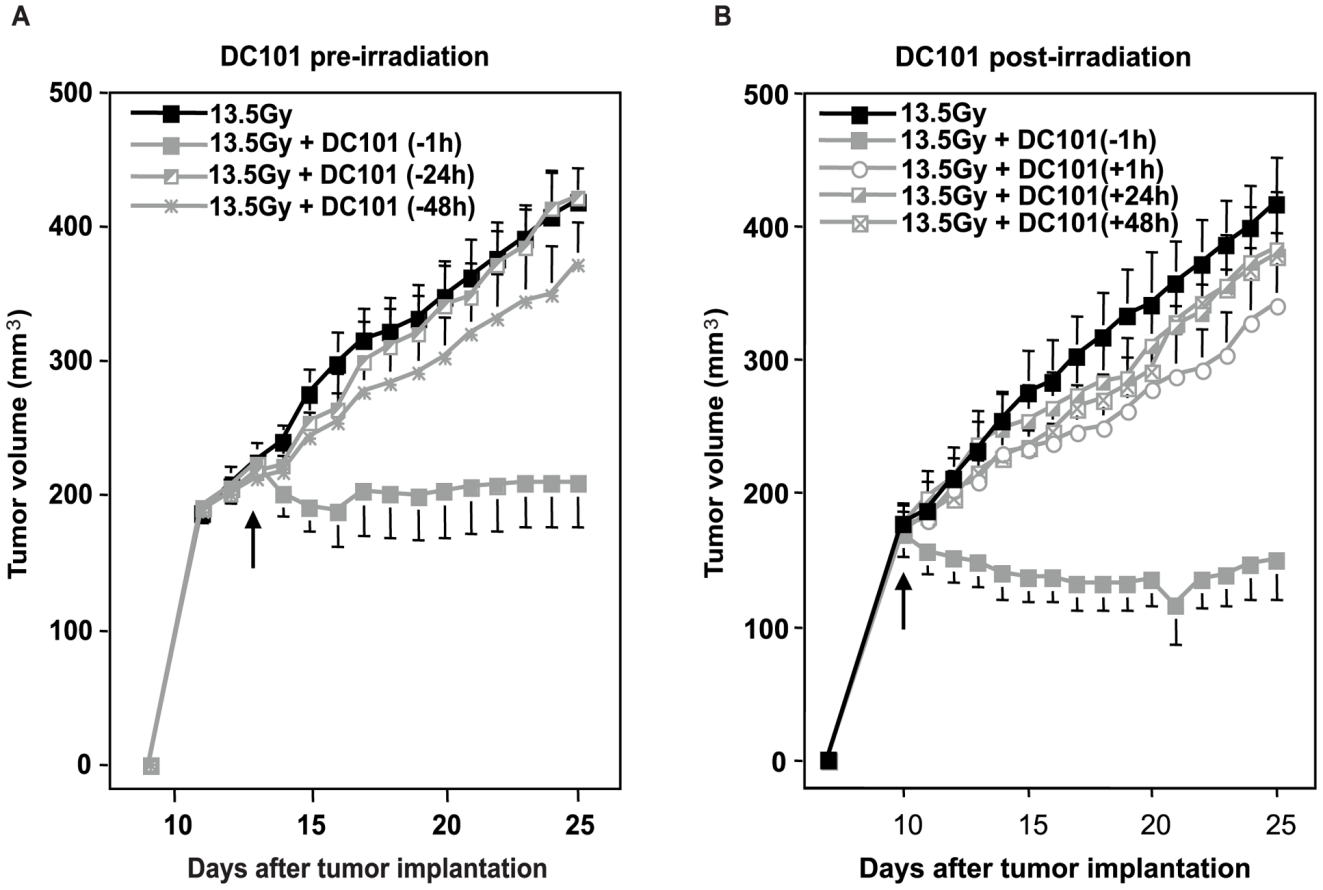
Impact of timing of DC101 addition on the radiation response of MCA/129-fibrosarcomas. DC101 (1600 µg/kg) was injected i.v. at the indicated times before (**A**) and after (**B**) irradiation. Data (means ±s.e.m.) were collated from 5 mice per group. Arrows indicate time of irradiation.

Whereas the *in vitro* studies showed that ASMase activation occurs within 1–2 min of radiation exposure of endothelial cells, we tested the timing of application of DC101 relative to radiation delivery. While DC101 pre-treatment at 1 h prior to radiation induced marked radiosensitization to a single high radiation dose of 13.5 Gy, DC101 injection 24 or 48 h prior to or at any time after radiation (from 1–48 h) was without effect (**Figure 6**). A more detailed kinetic analysis of the DC101 effect revealed optimal radiosensitization when it was delivered at 0.5–2 h pre-irradiation (data not shown). One or two doses of DC101 delivered intraperitoneally during the first week post-irradiation also had no further impact on tumor growth delay or tumor cure (data not shown). These studies indicate that anti-VEGF drugs need to be delivered shortly prior to radiation in order to radiosensitize, consistent with a requirement to neutralize VEGF within seconds to minutes after radiation, the time-window when the ceramide rheostat is activated to initiate the vascular component of single dose radiotherapy.

## Discussion

The present studies define a molecular mechanism for anti-angiogenic enhancement of tumor response to single dose radiotherapy via amplification of ceramide-driven endothelial apoptosis, converting transient tumor response to local cure. As such, the *in vivo* studies corroborate the current set of *in vitro* studies that identify the ambient ceramide level in the exoplasmic leaflet of the plasma membrane as dictating whether an angiogenic anti-apoptotic state or an anti-angiogenic pro-apoptotic state predominates. Both bFGF and VEGF suppress radiation-induced ASMase activation, ceramide generation and apoptosis, and restoration of ceramide levels by exogenous addition completely restores apoptosis even in continued presence of VEGF/bFGF. The ability to reverse the bFGF/VEGF effect by provision of the product of the enzymatic reaction regulated by ASMase definitively identifies ceramide, and not some other factor regulated by these pro-angiogenic growth factors, as conferring their anti-apoptotic potential. The studies thus indicate that radiation-induced apoptosis requires at least two signals, ceramide generation at the plasma membrane and a set of other, as yet unknown, events engaged upon radiation exposure. Furthermore, the fact that ceramide elevation “trumps” VEGF/bFGF suppression, and that it is requisite for anti-angiogenic radiosensitization, defines a ceramide rheostat that regulates the balance between endothelial cell survival and death, and ultimately tumor radiation response.

Although the current studies do not address the mechanism of radiation-induced translocation/activation of ASMase, a great deal of information has been published over the last decade that sheds light on this event. Diverse stresses (cytokines, heat, UV, ionizing radiation etc.) induce ASMase trafficking to the outer leaflet of the plasma membrane, where it finds its target sphingomyelin[20], [24], [25]. This process requires intact microtubules[26], phosphorylation of Ser508 of ASMase[27], as well as functional lipid rafts[28]. Ceramide generation in the outer plasma membrane leads to formation of ceramide-rich macrodomains (CRMs)[25], [26], that form within seconds to minutes of stress, are large (1–5 microns), and serve as sites of stress specific protein oligomerization[29], although for most stresses little is known about the specific multiprotein complexes that form therein. Although the precise target for VEGF inhibition of ASMase activation remains unknown, it is anticipated that it will be possible to make progress on this issue in future studies.

Perhaps the most important aspect of these studies is that use of anti-angiogenic agents as radiosensitizers *in vivo* was delineated based on the biology of radiation-induced generation of the pro-apoptotic second messenger ceramide in cultured endothelial cells. In this regard, the generation of ceramide within minutes of irradiation predicted that anti-angiogenic drugs should be delivered immediately prior to irradiation to de-repress ASMase acutely, yet not allow the system time to counterregulate, damping down the ceramide rheostat to a new level only slightly more sensitive than the original setting. Consistent with a need to deliver anti-angiogenic drugs just prior to irradiation is the empiric observation that pre-treatment of mice harboring tumors with DC101 at more than 2 h prior to or at any time after irradiation resulted in loss of radiosensitization. This temporal relationship between anti-angiogenic agents and radiation is inconsistent with the tumor microvessel normalization hypothesis, which requires at least 24 h to manifest[4], [30], and also with the proposed use of anti-angiogenics to prevent endothelial progenitor recruitment into the damaged site, usually delivered at 24 h prior to irradiation.

Combining radiation and anti-angiogenic radiosensitization may have practical clinical applications. Emerging evidence indicates that single dose radiotherapy, such as used in the present studies, may provide therapeutic benefit to some human tumors, even tumors considered resistant to conventional fractionated radiotherapy schemes. Early clinical experience with single dose radiotherapy in oligometastatic human cancers has demonstrated that local control of ∼90% can be achieved with single doses of 22–25 Gy[31]. These studies also showed that similar responses are achieved across a large variety of human tumor phenotypes, regardless of histologic classification. Other studies have employed three exposures of 12–20 Gy in treatment of a large variety of human cancers (the hypofractionated approach), demonstrating increasing local control rates as the dose per fraction escalated[32]. Because of the technical complexity associated with delivery of 24 Gy in a single session, there has been an ongoing search for tumor radiation sensitizers that would enable use of lower dose per fraction *via* currently affordable technologies, preserving the improved outcome provided by the higher exposures of single dose or hypofractionated schemes. In this context, it should be noted that several anti-angiogenic drugs have recently been developed for human use and are either in clinical use or currently being evaluated in clinical trials[1]. The present studies define a biologic basis for use of anti-angiogenic reagents in combination with high-dose (≥10 Gy) irradiation, given either as single dose or by the hypofractionated approach, providing an opportunity to further improve these emerging modalities. Specificity towards tumor versus normal tissue is currently realized by image-guided radiotherapy (IGRT) that targets the radiation beam onto the tumor with precision, nearly completely excluding surrounding normal tissues. Based on the principles defined in the present studies, clinical trials are currently being planned at MSKCC to employ anti-VEGF drugs as radiosensitizers, delivered immediately prior to radiotherapy. These studies are designed to explore the feasibility of de-escalating the dose levels of single dose-IGRT without compromising local tumor control.

## Materials and Methods

### Cell culture experiments

Cloned populations of bovine aortic endothelial cells (BAEC), obtained as previously described[33] and cultured in DMEM supplemented with 10% calf serum, 100 U/ml penicillin, 100 µg/ml streptomycin, and 2 mM L-glutamine at 37°C in a humidified 10% CO_2_ chamber. Unless otherwise stated, all other cell culture products were obtained through Mediatech. For experiments, BAEC were pre-incubated overnight in DMEM containing 0.2% human albumin. Human coronary aortic endothelial cells (HCAEC) were obtained from Cambrex and grown in EBM-2 medium with EGM-2 MV SingleQuot supplement (Cambrex) at 37°C in a humidified 5% CO2 chamber. For experiments HCAEC were pre-incubated in EBM-2 medium containing 0.5% FCS overnight. Irradiation of cultured cells was carried out in Shepherd Mark I irradiator containing a ^137^Cs source at a rate of 2.08 Gy/min. For experiments involving incubations for under 10 min, cells were irradiated on ice and thereafter warmed in a 37°C water bath for the indicated times. Basic FGF (Scios), rhVEGF-121 or rhVEGF-165 (R&D Systems) were added to cultures (1 ng/ml) 10 min before irradiation. For reconstitution of the ceramide effect, cells were pretreated with 1 µM C_16_-ceramide (Biomol) in dodecane:ethanol (2∶98, v/v; 0.05% final concentration) 30 min before irradiation. DC101 (kindly provided by ImClone), was added to cells 16 h before irradiation. Anti-ceramide IgM MAS0020 (Glycobiotech GmbH) was added 15 min before irradiation. To measure ASMase and ceramide levels at short time intervals, cells were irradiated on ice and incubated in a 37°C water bath for the indicated times according to published protocol[34].

### Mice and in vivo experiments

*asmase* ^+/+^ and *asmase* ^−/−^ mice, maintained in an sv129xBl/6 background, were propagated using heterozygous breeding pairs. Genotyping used a modification of the protocol described[35], employing a revised PA2 primer (5′-GGCTACCCGTGATATTGC-3′), and 35 cycles of PCR amplification, each at 94°C for 15 sec, 64°C for 30 sec, and 68°C for 90 sec. Mice were housed at the animal core facility of Memorial Sloan-Kettering Cancer Center. This facility is approved by the American Association for Accreditation of Laboratory Animal Care and is maintained in accordance with the regulations and standards of the United States Department of Agriculture and the Department of Health and Human Services, National Institutes of Health. The Institutional Animal Care and Use Committee (IACUC) at Memorial Sloan-Kettering Cancer Center approved the mice experiments under Protocol #92-10-038 on September 17, 2009.

MCA/129 fibrosarcoma and B16 melanoma cells were maintained in DMEM high glucose supplemented with 10% fetal calf serum (FCS), 100 U of penicillin/ml and 100 mg of streptomycin/ml in 10% CO_2_ at 37°C. Cells (10^6^), resuspended in PBS, and injected subcutaneously into the right flank as described[6]. Once tumors reached 100–150 mm^3^, mice were either treated intravenously or not with DC101 (1600 µg) or anti-VEGF mAb G6–31 (5 mg/kg, kindly provided by Genentech) or control IgG 1 h before irradiation. Radiation was delivered using a Philips MG-324 X-ray unit at 105.5 cGy/min (50 cm source to skin distance). Mice were lightly sedated with ketamine (0.1 mg/g) and xylazine (0.02 mg/g) and only tumor, surrounding skin and subcutaneous tissues, were exposed, the rest of the mouse was shielded using a specialized lead jig. Tumor volume, based on caliper measurements, was calculated daily according to the formula of Kim *et al*. [36].

### Quantification of Apoptosis

Morphologic changes in nuclear chromatin of cells undergoing apoptosis were detected by staining with the DNA-binding fluorochrome *bis*-benzimide trihydrochloride (Hoechst-33258), as described[37] using an Olympus BH2 fluorescence microscope equipped with a BH2-DMU2UV with Dich Mirror Cube filter. Alternately, caspase 3 activity was measured using fluorogenic AFC-tagged caspase-specific substrates purchased from Biovision, according to manufacturer's instructions.

To evaluate endothelial apoptosis *in vivo*, tumor samples were processed and stained as previously described[6]. Several different endothelial markers were evaluated for use on 5 µm paraffin embedded sections in combination with TUNEL; the best signal to noise ratio was achieved with a monoclonal antibody from GeneTex (Clone MEC14.7) and MECA-32 (Developmental Studies Hybridoma Bank, developed under the auspices of the NICHD and maintained by The University of Iowa, IA).

### Ceramide quantification

Upon termination of experiments, cells were washed once with cold PBS, and lipids were extracted using two 10 min incubations in 500 ml methanol at 4°C, followed by an equal volume of chloroform and 0.6 volume of buffered saline solution/EDTA solution (135 mM NaCl, 4.5 mM KCl, 1.5 mM CaCl_2_, 0.5 mM MgCl_2_, 5.6 mM glucose, 10 mM HEPES pH 7.2, 10 mM EDTA). Ceramide was quantified using the diacylglycerol kinase assay as described[38].

### ASMase Activity Measurement

ASMase activity was measured as described[39] with a few minor modifications. 3 mg of sample, including a control sample boiled for 20 min, were incubated with a mixture containing 2.5 nmol/ml of sphingomyelin (Indofine) and 3pCi of [*N-methyl*-^14^C]Sphingomyelin (Amersham) in 45 ml total volume. After a 1 h-incubation at 37°C, reactions were terminated with 100 ml buffer (CHCl_3_∶MeOH∶HCl, 100∶100∶1 v/v/v), centrifuged at 200×g, and 45 ml of the upper phase was removed and radioactivity measured by scintillation counting.

### Statistics

Values are expressed as mean±standard deviation unless otherwise noted. Paired, two-tailed students t tests were calculated using Prism v4. We considered P values less than 0.05 to be significant.

## Supporting Information

Methods S1 Supplementary Methods
(0.03 MB DOC)

Figure S1 bFGF inhibits radiation-induced ASMase translocation and ceramide generation. (A) Irradiated BAEC samples were collected at the indicated times and ASMase activity measured by quantifying conversion of [^14^C]sphingomyelin to the product [^14^C]phosphocholine. Data (mean±s.d.) represent duplicate determinations from 2 experiments. (B) Cells were fixed at the indicated times post-irradiation, stained with primary ASMase antibody (sc9815, 1∶10 v/v) and FITC-conjugated secondary Ab. 104 FITC-positive cells were counted by FACScan per point. Data (mean±s.d.) represent duplicate determinations from 2 experiments. (C) Ceramide was quantified at the indicated times after 10 Gy-irradiation by the DAG assay. bFGF was added 10 min before irradiation. Data (mean±s.d.) represent triplicate determinations from 2 experiments.
(1.56 MB TIF)

Figure S2 Ceramide generated in HCAEC in response to 20 Gy irradiation is inhibited by bFGF, VEGF-121 and -165. HCAEC were pre-incubated with 1 ng/ml of either bFGF,VEGF-121 or VEGF-165 10 min before 20 Gy irradiation. Ceramide was quantified at the indicated times using the diacylglycerol kinase method. Data (mean ±s.d.) are derived from triplicate determinations, representative of 2 independent experiments. Note that the Control and 20 Gy data are repeated in each panel for clarity.
(1.56 MB TIF)

Figure S3 VEGF-121 pre-treatment inhibits radiation-induced apoptosis. (A) C_16_-ceramide (1 µM) was added 30 min prior to irradiation, while VEGF-121 was added 10 min before. At the indicated times samples were fixed in 10% paraformaldehyde prior to *bis*-benzimide staining. Data (mean±s.d) represent duplicate determinations of at least 400 *bis*-benzimide stained nuclei counted from 2 experiments. (B) Caspase 3 activity was measured at the stated times after 10 Gy-irradiation by quantification of the luminescence of cleaved DEVD-AMC substrate. Data (mean±s.d.) represent duplicate points from 3 experiments.
(1.56 MB TIF)

Figure S4 bFGF pre-treatment inhibits radiation-induced apoptosis. C_16_-ceramide (1 µM) was added 30 min prior to irradiation, while bFGF (1 ng/ml) was added 10 min before. At the indicated times, the cells were fixed in 10% paraformaldehyde then stained with *bis*-benzimide before quantification of apoptotic nuclei. Data (mean±s.d) represent duplicate determinations of at least 400 *bis*-benzimide stained nuclei counted from 2 experiments.
(1.56 MB TIF)

Figure S5 bFGF, VEGF-121 and VEGF-165 protect HCAEC from radiation-induced apoptosis. HCAEC were prepared as described in the Supplementary Methods. C_16_-ceramide (0.4 µM) was added 30 min before irradiation while bFGF (A), VEGF-121 (B) or VEGF-165 (C), all at 1 ng/ml, were added 10 min before irradiation. Cells were fixed in 10% paraformaldehyde at the times shown and apoptosis was quantified by *bis*-benzimide staining. Data (mean±s.d.) represent duplicate determinations of at least 400 *bis*-benzimide stained nuclei collated from 2 experiments. Note that the Control and 20 Gy data are repeated in each panel for clarity.
(1.56 MB TIF)

Figure S6 bFGF pre-treatment inhibits radiation-induced caspase 3 activation. C_16_-ceramide (1 µM) was added 30 min while bFGF (1 ng/ml) was added 10 min prior to irradiation. Caspase 3 activity was measured by quantification of the luminescence of cleaved DEVD-AMC substrate. Data (mean±s.d.) represent duplicate points from 3 experiments.
(1.56 MB TIF)

Figure S7 Increasing bFGF does not overcome C_16_-ceramide restoration of radiation-induced apoptosis in BAEC. C_16_-ceramide (C_16_, 1 µM) was added 30 min before 10 Gy, while bFGF was added 10 min before. Apoptosis was quantified 8 h after irradiation at 10 Gy. Data (means±s.d.) are collated from at least 400 stained nuclei scored from duplicate points from 1 of 2 independent studies.
(1.56 MB TIF)

Figure S8 bFGF inhibits DC101-induced ceramide increase. bFGF was maximally effective at inhibiting ceramide generated by doses of DC101 up to 1 µg/ml. bFGF (1 ng/ml) was added 10 min before DC101, then ceramide was quantified 24 h later using the diacylglycerol kinase assay. Data (mean ±s.d.) are derived from triplicate determinations, representative of 2 independent experiments.
(1.56 MB TIF)

Figure S9 Escalating doses of bFGF do not further inhibit ceramide generated in DC101-treated BAEC. Escalating doses of bFGF were added 10 min before 5 µg/ml DC101. Ceramide was quantified 24 h later by *bis*-benzimide staining of nuclei. Data (mean±s.d.) are collated from triplicate determinations from 2 independent experiments.
(1.56 MB TIF)

Figure S10 Ceramide is required for radiation-induced apoptosis of HCAEC. HCAEC were incubated with 350 ng/ml of anti-ceramide Ab MAS0020 15 min before 20 Gy irradiation. Apoptotic nuclei were quantified after *bis*-benzimide staining at the time points shown. Data (means±s.d.) are collated from 2 independent studies and represent at least 400 stained nuclei scored from 4 points.
(1.56 MB TIF)

Figure S11 DC101 radiosensitization requires an apoptosis-sensitive vasculature. DC101 (1600 µg) was injected i.v. 1 h before 13.5 Gy irradiation of melanoma xenografts, grown in BAK^+/+^ or BAK^−/−^ mice that provide a radiosensitive or radioresistant vasculature, respectively. Tumor size was measured at the times shown. Data (means±s.e.m.) were collected from groups of 5 mice.
(1.56 MB TIF)

Figure S12 DC101 enhances radiation-induced endothelial cell apoptosis. Representative cross sections of MCA/129 fibrosarcomas stained with both an endothelial cell specific Ab (anti-CD34, blue) and TUNEL (brown).
(1.56 MB TIF)

Figure S13 Tumors grown in ASM^+/+^ and ASM^−/−^ mice display similar levels of endothelial cells. Endothelial cells, identified as being both CD31 and VEGFR2 expressing, were quantified by FACS analysis using disaggregated fibrosarcoma tumors prepared as described in the Supplemental Methods.
(1.56 MB TIF)
