# Correction: Endothelial Membrane Remodeling Is Obligate for Anti-Angiogenic Radiosensitization during Tumor Radiosurgery

**DOI:** 10.1371/annotation/6e222ad5-b175-4a00-9d04-4d120568a897

**Published:** 2010-09-30

**Authors:** Jean-Philip Truman, Mónica García-Barros, Matthew Kaag, Dolores Hambardzumyan, Branka Stancevic, Michael Chan, Zvi Fuks, Richard Kolesnick, Adriana Haimovitz-Friedman

This article was republished on September 29, 2010 because of illegible figures. Please download this article again to view the figures.

