# Supplementary material for: Endothelial Membrane Remodeling Is Obligate for Anti-Angiogenic Radiosensitization during Tumor Radiosurgery
Source: PLoS One. 2010 Aug 19;5(8):e12310. doi: 10.1371/journal.pone.0012310 (PMC2924400; doi:10.1371/journal.pone.0012310)
Supplement: Methods S1 — Supplementary Methods (0.03 MB DOC) [file pone.0012310.s001.doc]

**Supplementary Material for**

**Endothelial Membrane Remodeling is Obligate for**

**Anti-angiogenic Radiosensitization during Tumor Radiosurgery**

Jean-Philip Truman, Mónica García-Barros, Matthew Kaag, Dolores Hambardzumyan,

Branka Stancevic, Michael Chan, Zvi Fuks, Richard Kolesnick

and Adriana Haimovitz-Friedman*

*To whom correspondence should be addressed.

E-mail: a-haimovitz-friedman@ski.mskcc.org

Supporting Information includes:

Methods

Figures S1-S16

**Supplementary Methods**

**FACS analysis**

To obtain a single-cell suspension, tumors were dissected from hind limbs using a scalpel, washed twice in PBS, cut into small pieces and incubated in collagenase A cocktail (2mg/ml collagenase A (Roche), 250 mg/ml elastase (Roche), 25 mg/ml DNAse I (Roche) in DMEM supplemented with 1% FCS) at 37°C with gentle shaking. After 45 min, the tumor digest was filtered sequentially through 100 mm, 70 mm and 40 mm nylon filter mesh (BD Falcon). Cells were separated with Lympholyte-M (Cedarlane International), a density separation medium, to remove erythrocytes, dead cells and debris, at 1000-1500g for 20 min at room temperature. Cells were recovered from the interface. Single cell-suspensions were preincubated with purified anti-mouse CD16/CD32 (FcγIII/II Receptor) for 5 min and stained with PE anti-mouse Flk1 (VEGFR2) and FITC anti-mouse CD31 (PCAM-1) antibodies (BD Pharmingen) for 20 min at 4°C, and 10^4^ cells/sample were analyzed using a FACScan Cytometer (Becton Dickinson, Mountain View, CA). Data were analyzed using FlowJo 6.3.3 software.

**ASMase surface expression**

BAEC were resuspended in phosphate-buffered saline (PBS: KCl 0.2g/l, KH_2_PO_4_ 0.2g/l, NaCl 8.0g/l, Na_2_HPO_4_ 1.15g/l) at 4x10^6^/ml, irradiated, and incubated for the indicated times at 22°C prior to fixation in 4% paraformaldehyde. ASMase expression was measured by incubating PBS-washed cells with anti-ASMase Ab (polyclonal 1598 Ab)^20^ at 1:100 (v/v) dilution for 30 min. Thereafter, cells were washed with PBS, incubated with a donkey anti-goat FITC-conjugated secondary Ab (Jackson Immunochemicals) for 30 min, re-washed and analyzed by FACS. Data were analyzed using FlowJo version 6 software.

**Tumor staining**

To evaluate endothelial apoptosis, tumor samples were processed and stained as previously described. Several different endothelial markers were evaluated for use on 5 µm paraffin embedded sections in combination with TUNEL; the best signal to noise ratio was achieved with a monoclonal anti-CD34 antibody from GeneTex (Clone MEC14.7).
