# Supplementary figures and images for: Endothelial Membrane Remodeling Is Obligate for Anti-Angiogenic Radiosensitization during Tumor Radiosurgery
Source: PLoS One. 2010 Aug 19;5(8):e12310. doi: 10.1371/journal.pone.0012310 (PMC2924400; doi:10.1371/journal.pone.0012310)

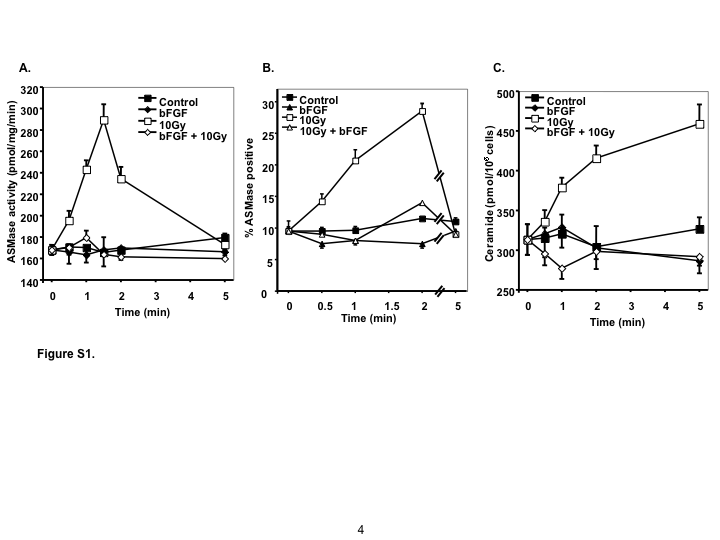

Supplement: Figure S1 — bFGF inhibits radiation-induced ASMase translocation and ceramide generation. (A) Irradiated BAEC samples were collected at the indicated times and ASMase activity measured by quantifying conversion of [14C]sphingomyelin to the product [14C]phosphocholine. Data (mean±s.d.) represent duplicate determinations from 2 experiments. (B) Cells were fixed at the indicated times post-irradiation, stained with primary ASMase antibody (sc9815, 1∶10 v/v) and FITC-conjugated secondary Ab. 104 FITC-positive cells were counted by FACScan per point. Data (mean±s.d.) represent duplicate determinations from 2 experiments. (C) Ceramide was quantified at the indicated times after 10 Gy-irradiation by the DAG assay. bFGF was added 10 min before irradiation. Data (mean±s.d.) represent triplicate determinations from 2 experiments. (1.56 MB TIF) [file pone.0012310.s002.tif]

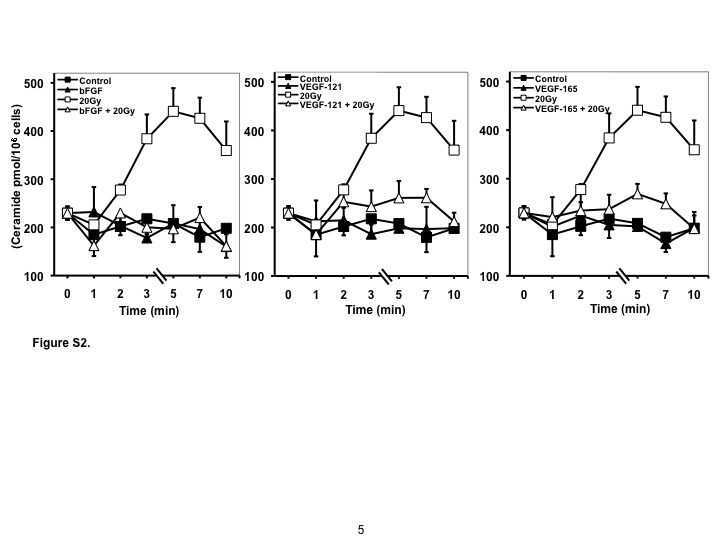

Supplement: Figure S2 — Ceramide generated in HCAEC in response to 20 Gy irradiation is inhibited by bFGF, VEGF-121 and -165. HCAEC were pre-incubated with 1 ng/ml of either bFGF,VEGF-121 or VEGF-165 10 min before 20 Gy irradiation. Ceramide was quantified at the indicated times using the diacylglycerol kinase method. Data (mean ±s.d.) are derived from triplicate determinations, representative of 2 independent experiments. Note that the Control and 20 Gy data are repeated in each panel for clarity. (1.56 MB TIF) [file pone.0012310.s003.tif]

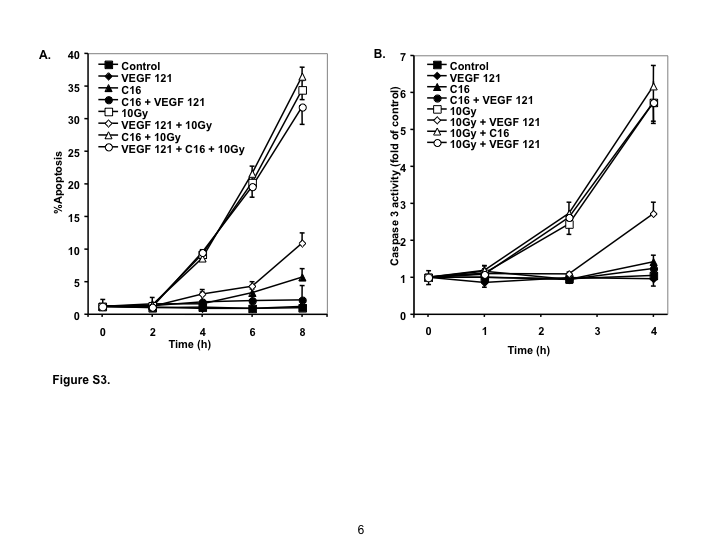

Supplement: Figure S3 — VEGF-121 pre-treatment inhibits radiation-induced apoptosis. (A) C16-ceramide (1 µM) was added 30 min prior to irradiation, while VEGF-121 was added 10 min before. At the indicated times samples were fixed in 10% paraformaldehyde prior to bis-benzimide staining. Data (mean±s.d) represent duplicate determinations of at least 400 bis-benzimide stained nuclei counted from 2 experiments. (B) Caspase 3 activity was measured at the stated times after 10 Gy-irradiation by quantification of the luminescence of cleaved DEVD-AMC substrate. Data (mean±s.d.) represent duplicate points from 3 experiments. (1.56 MB TIF) [file pone.0012310.s004.tif]

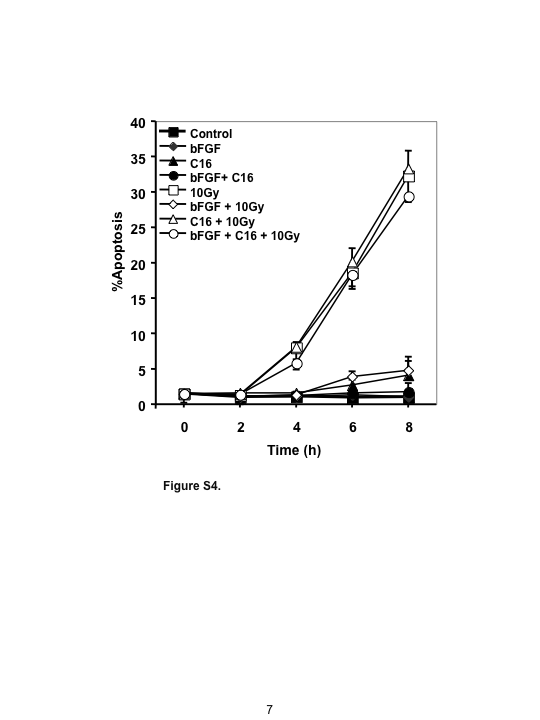

Supplement: Figure S4 — bFGF pre-treatment inhibits radiation-induced apoptosis. C16-ceramide (1 µM) was added 30 min prior to irradiation, while bFGF (1 ng/ml) was added 10 min before. At the indicated times, the cells were fixed in 10% paraformaldehyde then stained with bis-benzimide before quantification of apoptotic nuclei. Data (mean±s.d) represent duplicate determinations of at least 400 bis-benzimide stained nuclei counted from 2 experiments. (1.56 MB TIF) [file pone.0012310.s005.tif]

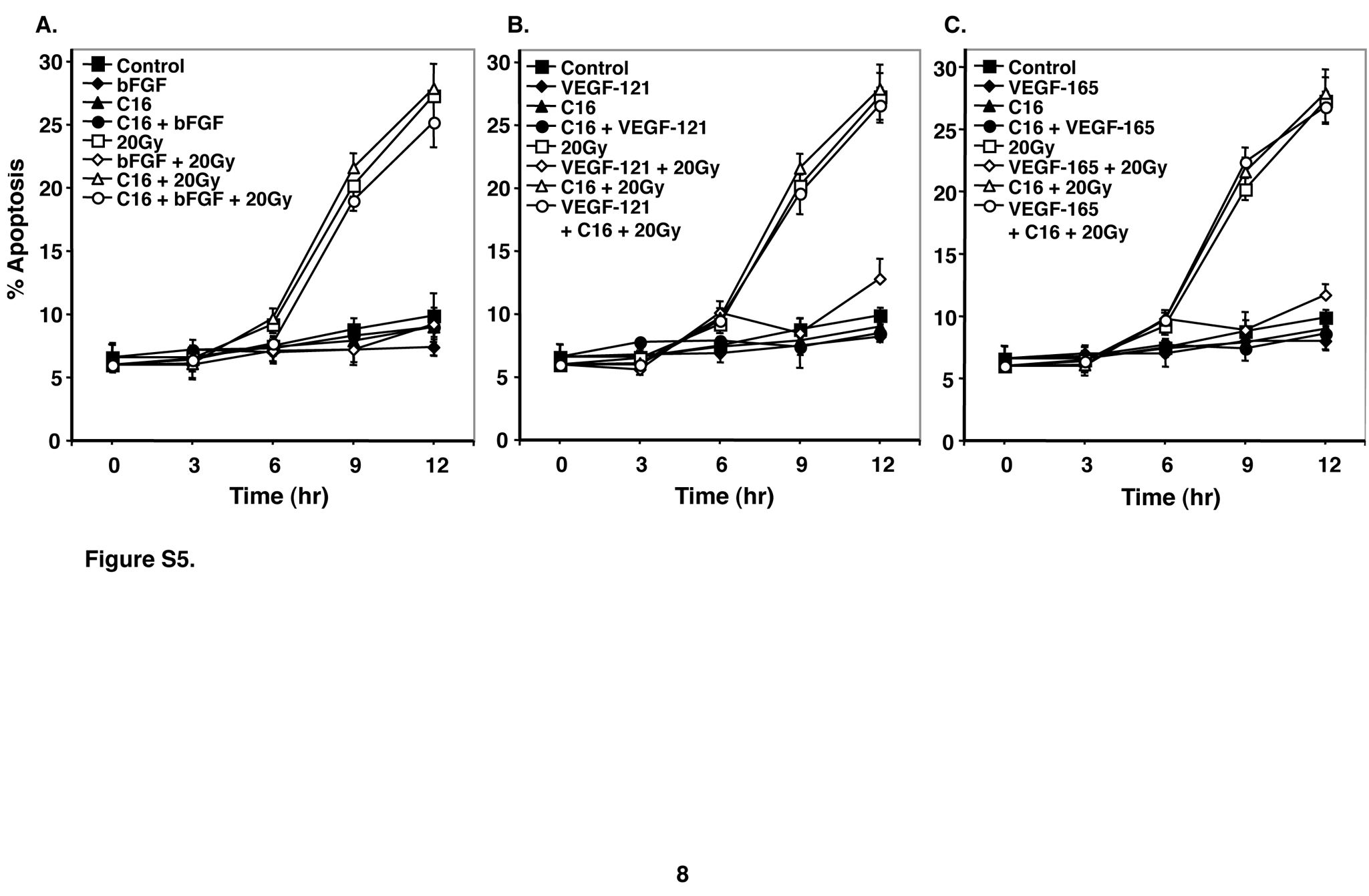

Supplement: Figure S5 — bFGF, VEGF-121 and VEGF-165 protect HCAEC from radiation-induced apoptosis. HCAEC were prepared as described in the Supplementary Methods. C16-ceramide (0.4 µM) was added 30 min before irradiation while bFGF (A), VEGF-121 (B) or VEGF-165 (C), all at 1 ng/ml, were added 10 min before irradiation. Cells were fixed in 10% paraformaldehyde at the times shown and apoptosis was quantified by bis-benzimide staining. Data (mean±s.d.) represent duplicate determinations of at least 400 bis-benzimide stained nuclei collated from 2 experiments. Note that the Control and 20 Gy data are repeated in each panel for clarity. (1.56 MB TIF) [file pone.0012310.s006.tif]

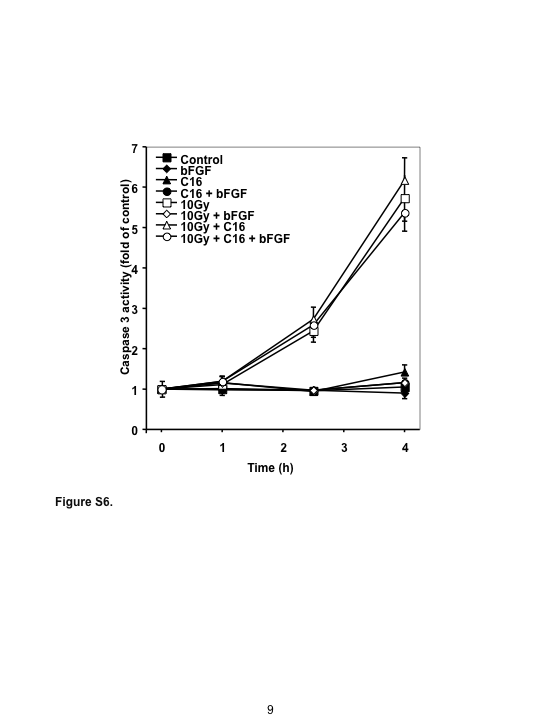

Supplement: Figure S6 — bFGF pre-treatment inhibits radiation-induced caspase 3 activation. C16-ceramide (1 µM) was added 30 min while bFGF (1 ng/ml) was added 10 min prior to irradiation. Caspase 3 activity was measured by quantification of the luminescence of cleaved DEVD-AMC substrate. Data (mean±s.d.) represent duplicate points from 3 experiments. (1.56 MB TIF) [file pone.0012310.s007.tif]

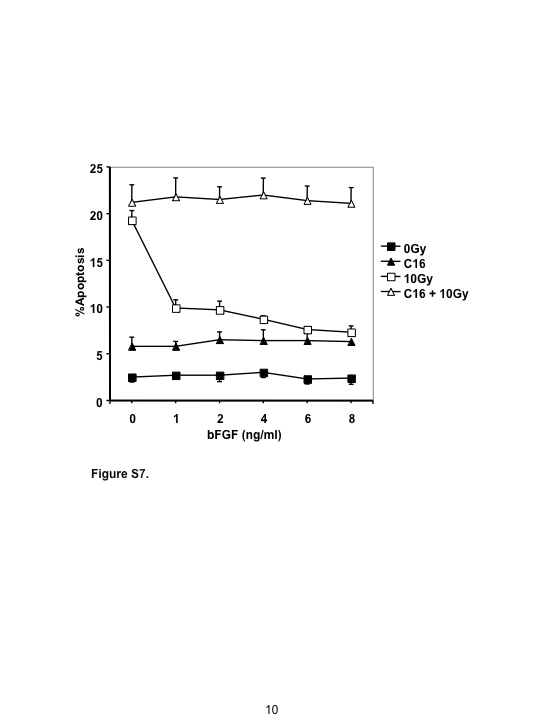

Supplement: Figure S7 — Increasing bFGF does not overcome C16-ceramide restoration of radiation-induced apoptosis in BAEC. C16-ceramide (C16, 1 µM) was added 30 min before 10 Gy, while bFGF was added 10 min before. Apoptosis was quantified 8 h after irradiation at 10 Gy. Data (means±s.d.) are collated from at least 400 stained nuclei scored from duplicate points from 1 of 2 independent studies. (1.56 MB TIF) [file pone.0012310.s008.tif]

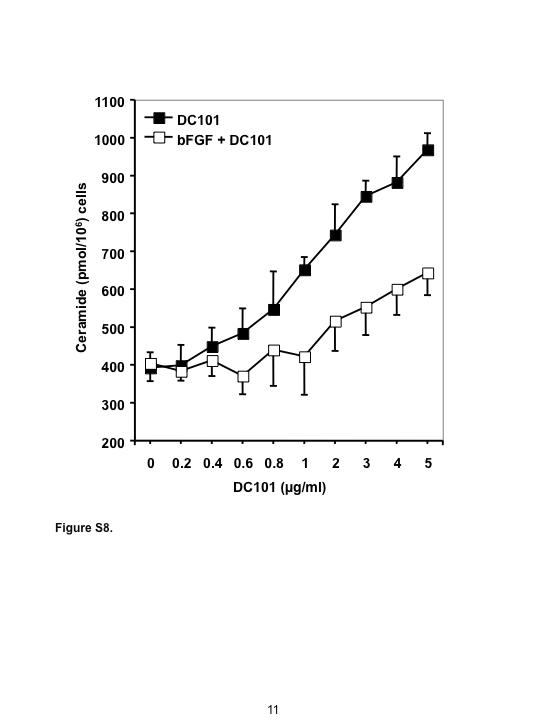

Supplement: Figure S8 — bFGF inhibits DC101-induced ceramide increase. bFGF was maximally effective at inhibiting ceramide generated by doses of DC101 up to 1 µg/ml. bFGF (1 ng/ml) was added 10 min before DC101, then ceramide was quantified 24 h later using the diacylglycerol kinase assay. Data (mean ±s.d.) are derived from triplicate determinations, representative of 2 independent experiments. (1.56 MB TIF) [file pone.0012310.s009.tif]

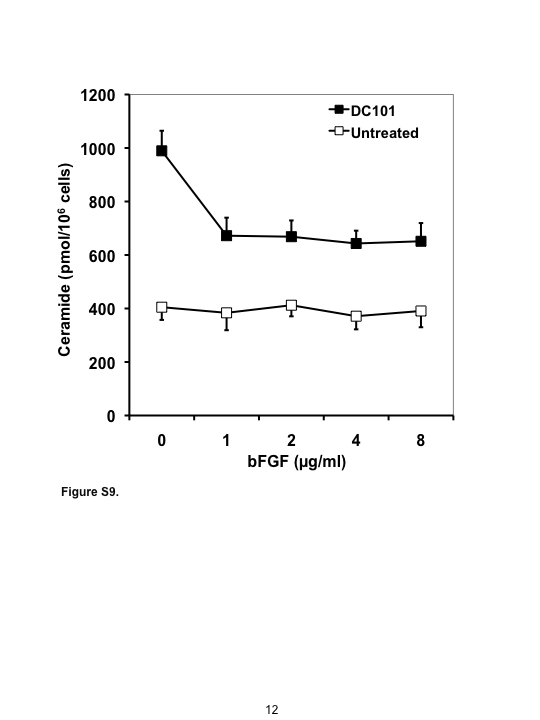

Supplement: Figure S9 — Escalating doses of bFGF do not further inhibit ceramide generated in DC101-treated BAEC. Escalating doses of bFGF were added 10 min before 5 µg/ml DC101. Ceramide was quantified 24 h later by bis-benzimide staining of nuclei. Data (mean±s.d.) are collated from triplicate determinations from 2 independent experiments. (1.56 MB TIF) [file pone.0012310.s010.tif]

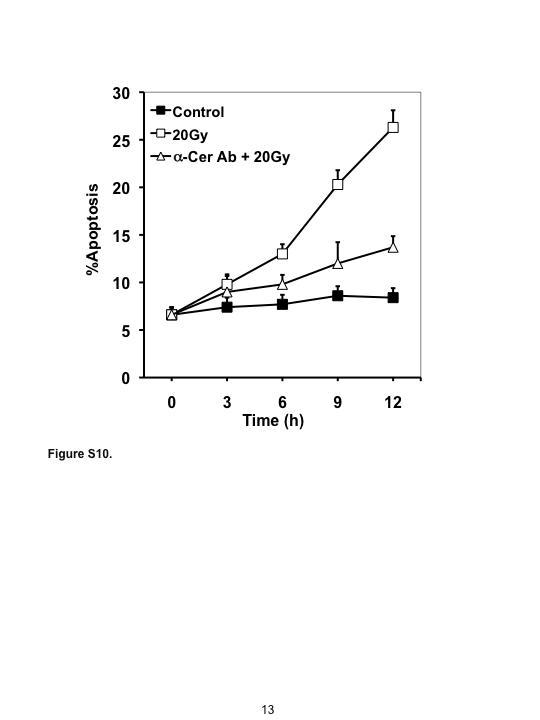

Supplement: Figure S10 — Ceramide is required for radiation-induced apoptosis of HCAEC. HCAEC were incubated with 350 ng/ml of anti-ceramide Ab MAS0020 15 min before 20 Gy irradiation. Apoptotic nuclei were quantified after bis-benzimide staining at the time points shown. Data (means±s.d.) are collated from 2 independent studies and represent at least 400 stained nuclei scored from 4 points. (1.56 MB TIF) [file pone.0012310.s011.tif]

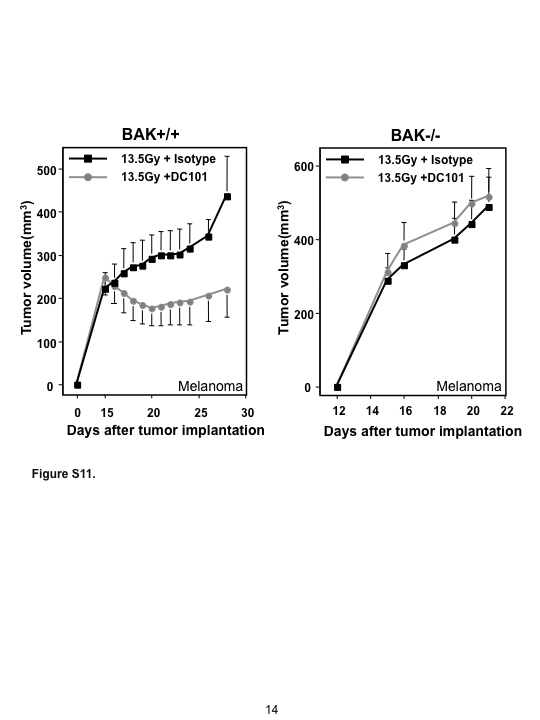

Supplement: Figure S11 — DC101 radiosensitization requires an apoptosis-sensitive vasculature. DC101 (1600 µg) was injected i.v. 1 h before 13.5 Gy irradiation of melanoma xenografts, grown in BAK+/+ or BAK−/− mice that provide a radiosensitive or radioresistant vasculature, respectively. Tumor size was measured at the times shown. Data (means±s.e.m.) were collected from groups of 5 mice. (1.56 MB TIF) [file pone.0012310.s012.tif]

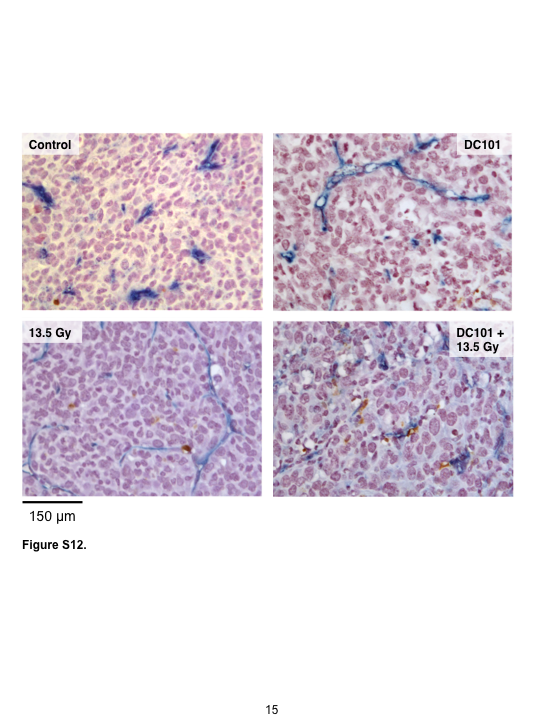

Supplement: Figure S12 — DC101 enhances radiation-induced endothelial cell apoptosis. Representative cross sections of MCA/129 fibrosarcomas stained with both an endothelial cell specific Ab (anti-CD34, blue) and TUNEL (brown). (1.56 MB TIF) [file pone.0012310.s013.tif]

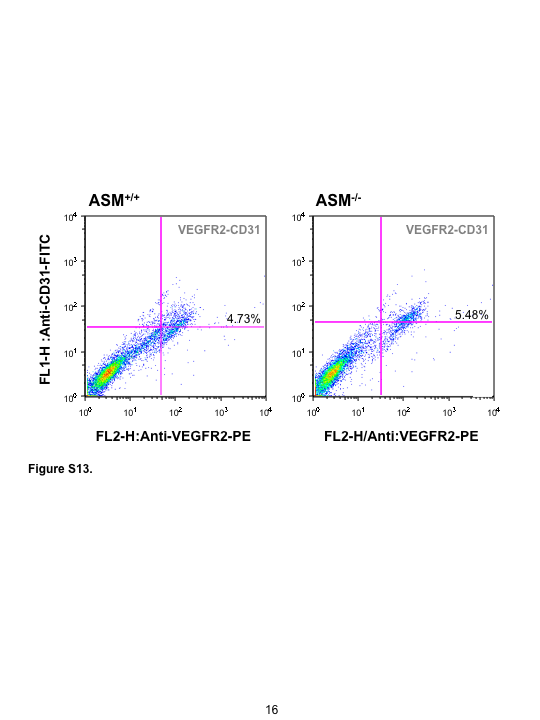

Supplement: Figure S13 — Tumors grown in ASM+/+ and ASM−/− mice display similar levels of endothelial cells. Endothelial cells, identified as being both CD31 and VEGFR2 expressing, were quantified by FACS analysis using disaggregated fibrosarcoma tumors prepared as described in the Supplemental Methods. (1.56 MB TIF) [file pone.0012310.s014.tif]
